# Treatment strategies for treatment naïve HIV patients in Germany: evidence from claims data

**DOI:** 10.1186/s40064-015-1099-z

**Published:** 2015-07-01

**Authors:** Jörg Mahlich, Johannes R Bogner, Jörg Tomeczkowski, Matthias Stoll

**Affiliations:** Janssen K.K., Tokyo, Japan; Düsseldorf Institute for Competition Economics (DICE), Heinrich-Heine University of Düsseldorf, Düsseldorf, Germany; Med. Klinik und Poliklinik IV, Sektion Klinische Infektiologie, University Hospital of Munich, Munich, Germany; Janssen-Cilag, Neuss, Germany; Hannover Medical School (MHH), Hannover, Germany

**Keywords:** HIV infection, AIDS, NNRTI, PI, Antiretroviral treatment, Decision, Real world evidence, Claims data

## Abstract

A recent observational study of HIV patients in Germany suggests that treatment naïve patients that are in a more advanced stage of their disease are more likely to receive a treatment regimen based on a boosted protease inhibitor (PI/r) compared with a non-nucleoside reverse-transcriptase-inhibitor (NNRTI) base regimen. To validate those results we analysed claims data of seven German sickness funds from 2009 to 2012 with approximately 4 million beneficiaries. Patients in a more advanced disease state (CDC class C) had a higher likelihood to receive a PI/r based regime rather than a NNRTI based regimen as their initial treatment. There was also a significant correlation between PI/r based regimen and number of comorbidities but not with age. Our results confirm a highly significant relationship between being in a more severe stage of HIV disease and a PI/r based treatment regimen.

## Background

A recent analysis of a German cohort of human immunodeficiency virus (HIV) infected patients revealed that the choice of the treatment regimen for the initiation of antiretroviral therapy is not random. Rather, the results of the study suggest that patients that are in a more severe stage in HIV do more likely receive a boosted protease inhibitor (PI/r) based treatment regimen compared with a non-nucleoside reverse-transcriptase-inhibitor (NNRTI) based regimen (Mahlich et al. 2015). The rationale for this treatment decision can be seen in the different resistance barriers of the two drug classes. Previous research found that impaired adherence has a bigger impact on treatment failure in NNRTI-based as compared to PI/r-based treatment strategies (Parienti et al. 2010; Rosenbloom et al. 2012). To avoid antiretroviral resistance and subsequent virological failure, patients that are believed to take their medication only irregularly would preferably receive a PI/r based regimen, while patients with a potentially good adherence may receive a NNRTI based regimen. The obvious question is then, how physicians can identify patient’s future adherence a priory. Some determinants of adherence have been identified in the literature that might provide some guidance to the physician. Identified factors that negatively influence adherence include lower age (Hinkin et al. 2004), lower income (Carballo et al. 2004), concomitant diseases (Shah et al. 2007), as well as disease specific factors such as an advanced disease stage (Protopopescu et al. 2009). Using the CDC classification system for HIV-infection (Centers for Disease Control and Prevention 1992), the German observational study mentioned before (Mahlich et al. 2015) established a relationship between the likelihood of PI/r prescription and CDC status C which indicates the worst disease status (CDC status A on the other hand would indicate the mildest form of the disease). The goal of this study is to validate the findings of the observational study with German claims data. Permission was granted to access the data and the analysis was carried out according to the guidelines of all institutions involved.

## Methods

We analysed claims data of seven German sickness funds from 2009 to 2012 with approximately 4 million insurants. 5,792 or 0.14% of them were found to be diagnosed with HIV, we refer to them as ‘people living with HIV or AIDS’ (PLWHA). Only 35.6% of the diagnosed patients receive antiretroviral treatment of which 21.2% were identified as being on their first treatment regimen (Table 1). The low treatment rate can be attributed to coding errors that have inflated the number of HIV diagnoses in Germany during our observation period (Tomeczkowski et al. 2015). As we only consider patients under treatment, our analysis is not affected by this bias. A patient was defined to be on first line treatment regimen when she has received no prior treatment. The minimum observation period to determine the treatment status was 6 month. That is to say that in order to qualify for the treatment naïve status a patient was at least for 6 month without antiretroviral prescriptions. Applying this definition we might have incorrectly defined a treatment experienced patient as being treatment naïve when this patient is on a treatment break of more than 6 month. As treatment breaks are not recommended in any guidelines we do not believe that this source of a potential bias is significant.

**Table 1:** Description of the sample

				Number of patients (%)
Total sick fund population				4,000,000 (100)
HIV diagnosed	Total PLWHA			5,792 (0.14)
	ART	Total ART		2,082 (35.95)
		Tx naive	Total Tx naive	441 (21.18)
			PI-based	174 (39.46)
			NNRTI-based	141 (31.97)
			Others	126 (28.57)

The disease stage according to the CDC classification system is not recorded in the claims database. To test the proposition that the treatment regimen is related to the disease stage, we therefore had to construct the CDC classification based on co-morbidities. CDC stage defining co-morbidities can be found in a publication of the U.S. Department of Health and Human Services (2014) and are reported in the “Appendix”.

We then analyse if the distribution of patients in disease stage CDC C differs across the two treatment strategies (PI/r and NNRTI based regimen). To check the significance of any observed difference we apply a Chi squared test. The p value <0.5 (two sided) was considered as being statistically significant.

We also compare our result with that of Mahlich et al. (2015) which is based on observational data. We are able to make this comparison also with regards to two other patient characteristics, namely ‘age’ and having ‘three or more concomitant diseases’.

## Results

**Figure 1:**
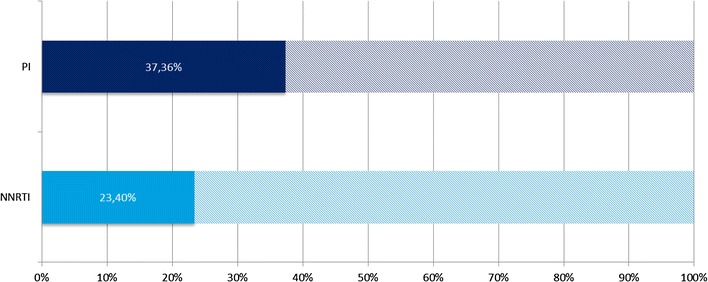
Share of treatment naïve patients in CDC C stage according to treatment regimen.

The result of our analysis is displayed in Figure 1 that shows the proportion of treatment naïve CDC C patients according to the drug class. It can be seen from the chart that 37.4% of the treatment naïve PI/r patients are in CDC stage C while this fraction is only 23.4% for the patients receiving a NNRTI based treatment regimen. The Chi squared test takes the value 15.224 and the associated p value is <0.001. The observed difference is therefore highly significant.

In Table 2 the results with regard to ‘age’ and ‘three or more concomitant disease’ are compared with those of Mahlich et al. (2015). With regard to age we found no significant difference between NNRTI and PI/r patients (p value 0.139) which is also the result reported by the German observational study. On the other hand we found a significant difference with regard to the presence of more than concomitant diseases. Only 27.6% of NNRTI patients had three or more concomitant diseases, while this fraction was 37.1% for PI/r patients. The difference was statistically significant as well (p value 0.050), implying that multi morbid patients are more likely to receive a PI/r based treatment.

**Table 2:** Claims data vs. observational data

	NNRTI		PI	
	Claim data (%)	Observational data (Mahlich et al. 2015) (%)	Claim data (%)	Observational data (Mahlich et al. 2015) (%)
Socio-demographic factors				
Patient age (in years) at diagnosis				
<50	62.8	8.3	67.2	89.5
≥50	37.2	21.7	32.8	10.5
Anamnestic factors				
HIV stage according to CDC-classification				
A + B	76.6	89.1	62.9	63.2
C (aids)	23.4	10.9	37.1	36.8
Three or more concomitant diseases				
No	72.7	80.4	62.9	76.3
Yes	27.6	19.6	37.1	23.7

## Discussion

The results presented in this paper confirm that physicians´ treatment decision towards a PI/r based treatment strategy for the initiation of antiretroviral treatment in therapy naïve HIV patients is influenced by a more advanced disease stage of HIV-infection. Contrary to the results of the German observational study we also found the number of concomitant diseases significantly related to a PI/r based treatment regimen. Despite today’s effective and available antiretroviral treatment, a significant proportion of PLWHA are still diagnosed as late presenters in progressed disease stages. This particular subgroup is characterized by a worse outcome and causes higher costs to the health care system. Both health policy decision makers and physicians should certainly consider improved strategies to address individuals with high risk to prevent late presentation.

To our knowledge this analysis investigating parameters that drive the treatment decision between NNRTI and PI/r based regimens for the initiation of antiretroviral therapy is the first study using claims data. So far this research question has only be analysed in the context of observational studies, be it in the UK (Easterbrook et al. 2008), Switzerland (Elzi et al. 2012) or Germany (Mahlich et al. 2015). The results also highlight the opportunities that arise with the utilization of claims data which are increasingly easy to access.

## Conclusions

The German claims data analysis confirms that the treatment decision for NNRTI or PI/r based regimen is associated with the disease severity. The result is in line with results from observational studies.

## Appendix

See Table 3.

**Table 3:** List of comorbidities defining CDC stage B and C

Category B
Bacillary angiomatosis
Oropharyngeal Candida infection
Vulvovaginal candida infections, which are either chronic (longer than 1 month) or only poorly treatable
Cervical dysplasia or carcinoma in situ
Constitutional symptoms such as fever above 38.5°C or more than 4 weeks existing diarrhea
Oral hairy leukoplakia
Herpes zoster infection in multiple dermatomes or after relapse in a dermatome
Idiopathic thrombocytopenic purpura
Listeriosis
Inflammation of the pelvis, especially when complications of tubal or Ovarialabszesses
Peripheral neuropathy

Category C
*Pneumocystis jirovecii* pneumonia
Toxoplasma encephalitis
Oesophageal candida infection or infection of the bronchi, trachea or lungs
Chronic herpes simplex or herpes ulcers bronchitis, pneumonia or esophagitis
CMV retinitis
Generalized CMV infection (not liver or spleen)
Recurrent *Salmonella* septicemia
Recurrent pneumonias within one year
Extrapulmonary cryptococcal infections
Chronic intestinal cryptosporidiosis infection
Chronic intestinal infection with Isospora belli
Disseminated histoplasmosis or extrapulmonary
Tuberculosis
Infections with mycobacterium avium complex or M. kansasii, disseminated or extrapulmonary
Kaposi’s sarcoma
Malignant lymphomas (Burkitt, immunoblastic or primary cerebral lymphoma)
Invasive cervical carcinoma
HIV encephalopathy
Progressive multifocal leukoencephalopathy
Wasting syndrome
